# Prevalence and socio-economic determinates of food insecurity in Veterans: findings from National Health and Nutrition Examination Survey

**DOI:** 10.1017/S1368980023000538

**Published:** 2023-07

**Authors:** Ronna Robbins, Kathryn N Porter Starr, Odessa Addison, Elizabeth A Parker, Sarah J Wherry, Sunday Ikpe, Monica C Serra

**Affiliations:** 1 Department of Nutritional Sciences, University of Texas at Austin, Austin, TX 78712, USA; 2 San Antonio GRECC, South Texas Veterans Health Care System, San Antonio, TX 78229, USA; 3 Durham GRECC, Durham VA Health Care System, Durham, NC, USA; 4 Division of Geriatrics, Duke University School of Medicine, Durham, NC, USA; 5 Baltimore GRECC, VA Maryland Health Care System, Baltimore, MD, USA; 6 Department of Physical Therapy and Rehabilitation Science, University of Maryland School of Medicine, Baltimore, MD, USA; 7 VA Eastern Colorado GRECC, Aurora, CO, USA; 8 Division of Geriatric Medicine, University of Colorado Anschutz Medical Campus, Aurora, CO, USA; 9 Division of Geriatrics, Gerontology and Palliative Medicine and the Sam and Ann Barshop Institute for Longevity and Aging Studies, Department of Medicine, UT Health San Antonio, San Antonio, TX, USA

**Keywords:** Veteran, National Health and Nutrition Examination Survey, Food insecurity, Social determinants

## Abstract

**Objective:**

To determine predictors of the association between being a Veteran and adult food security, as well as to examine the relation of potential covariates to this relationship.

**Design:**

Data collected during 2011–2012, 2013–2014 and 2015–2016 National Health and Nutrition Examination Survey (NHANES) were pooled for analyses. Veterans (self-reported) were matched to non-Veterans on age, race/ethnicity, sex and education. Adjusted logistic regression was used to determine the odds of Veterans having high food security *v*. the combination of marginal, low and very low food security compared with non-Veterans.

**Setting:**

2011–2012, 2013–2014 and 2015–2016 NHANES.

**Participants:**

1227 Veterans; 2432 non-Veterans.

**Results:**

Veteran status had no effect on the proportion of food insecurities between Veterans and non-Veterans reporting high (Veterans *v*. non-Veteran: 79 % *v*. 80 %), marginal (9 % *v*. 8 %), low (5 % *v*. 6 %) and very low (8 % *v*. 6 %) food security (*P* = 0·11). However, after controlling for covariates, Veterans tended to be less likely to have high food security (OR: 0·82 (95 % CI 0·66, 1·02), *P* = 0·07). Further, non-Hispanic White Veterans (OR: 0·72 (95 % CI 0·55, 0·95), *P* = 0·02) and Veterans completing some college (OR: 0·71 (95 % CI 0·50, 0·99), *P* < 0·05) were significantly less likely to experience high food security compared with non-Veterans.

**Conclusion:**

This study supports previous research findings that after controlling for covariates, Veterans tend to be less likely to have high food security. It also highlights ethnicity and level of education as important socio-economic determinates of food security status in Veterans.

Households that are food secure have access at all times to adequate food to stay healthy, whereas food insecurity, or the limited or uncertain access to adequate and appropriate food, is a multifaceted phenomenon independently associated with chronic health conditions^(1)^, mobility limitations^(2)^ and poor overall health^(3)^. Food insecurity also highlights another area of concern – a choice between paying for food and paying for medication. This dilemma leads to a higher risk for uncontrolled chronic health conditions and increased healthcare costs because of an inability to adhere to disease-specific diet modifications and medication treatment plans^(4)^.

Disability has been identified as a risk factor for food insecurity, such that households with an adult unable to work due to a physical or mental health disability are three times more likely to experience food insecurity^(5)^. Despite being known for their resiliency during and following their military service, Veterans are more likely than non-Veterans to be disabled because of their unique exposures during military service^(6)^. Furthermore, both male and female Veterans are significantly more likely to have multiple chronic health conditions compared with their non-Veteran counterparts^(7)^. Having multiple chronic conditions is also associated with food insecurity in younger and older adults^(3)^. Despite these risk factors suggesting Veterans are more vulnerable to food insecurity the findings regarding food insecurity in Veterans are currently inconsistent with reports of higher^(8)^, lower^(9)^ and equivocal^(10)^ findings of food insecurity in the literature. Reports from the Current Population Survey Food Security Supplement for 2005–2013 outlined lower rates of food insecurity between Veterans (8·4%) and non-Veterans (14·4%)^(11)^, and pooled data from the 2012 Health and Retirement Study and 2013 Health Care and Nutrition Mail Survey reported similar rates of food insecurity between Veterans (6·4%) and non-Veterans (11·9 %)^(9)^. However, a recent 2021 economic research report by the US Department of Agriculture found that after controlling for undefined ‘observable differences’ in socio-economic, demographic and military characteristics, working-aged Veterans (18–64 years) were 7·4% more likely to live in a food insecure household than non-Veterans^(8)^. These data highlight the need for further evaluation to understand the influence of prior military service on food security status.

With a growing body of evidence finding socio-economic and biomedical differences between Veterans and non-Veterans (i.e. poverty, healthcare utilisation, comorbid conditions), it is important to examine the impact these differences may have on food security status^(8)^, since many have been linked to food security status previously^(12–15)^. The understanding of socio-economic and biomedical factors associated with food security status in Veterans will aid in the development of interprofessional interventions and partnerships in clinical and community settings targeted to address the unique needs of the Veteran population.

The National Health and Nutrition Examination Survey (NHANES), a programme of studies comprised of questionnaires and physical clinical examinations used to assess the health of a national representative sample of Americans, is another source of potential data to examine this relationship. A recent NHANES study examined the risk for low and very low food security between socio-economically matched Veterans and non-Veterans and found that the prevalence was similar between groups (17·4 % Veterans and 16·7 % non-Veterans) and that Veteran status did not increase the odds of food insecurity in unadjusted or adjusted (age, gender, race/ethnicity, education, marital status, family income-to-poverty ratio and depression) analyses^(10)^. However, these analyses were limited to Veterans of working age with children. The exclusion of older Veterans is important to note, given that older adults are one of the fastest growing populations, and Veterans represent a significant proportion of this ageing population^(16)^. Older adults and particularly older Veterans may experience additional financial barriers that may influence food intake such as limited income due to retirement or an inability to work caused by one or more chronic health conditions^(17)^; thus, the results cannot be generalised to the Veteran population as a whole. Therefore, the objective of this study was to utilise NHANES to determine predictors of the association between being a Veteran and adult food security, as well as to examine the relation of potential covariates to this relationship.

## Methods

### Study design and sample

NHANES data collected during 2011–2012, 2013–2014 and 2015–2016 were pooled for analyses. A detailed overview of NHANES data collection can be viewed in the NHANES Lab Procedures Manual available at www.cdc.gov/nchs/nhanes/index.htm. Specific to this study, we included only those self-reporting either ‘yes’ or ‘no’ as to whether they had ‘ever served on active duty in the US Armed Forces, military Reserves, or National Guard’. Those answering ‘yes’ were classified as Veterans, and those answering ‘no’ were classified as non-Veterans. During the household interview, adults responded to the ten questions of the US Food Security Survey Module (US FSSM) (see Table 1)^(18)^, and level of adult food security was coded by NHANES study staff on the following responses to the US FSSM:

1. High/full: no affirmative response to any question
2. Marginal: 1–2 affirmative responses
3. Low: 3–5 affirmative responses
4. Very low: 6–10 affirmative responses.

**Table 1:** US Food Security Survey Module (FSSM) for households without children by Veteran status from National Health and Nutrition Examination Survey (NHANES) 2011–16

Question	Veteran		non-Veteran		Response options
	*n*	%	*n*	%	
In the last 12 months:	Affirmative/total response				
1) {I/we} worried whether {my/our} food would run out before {I/we} got money to buy more	271/1227	22 %	605/2432	25 %	**Often true** **Sometimes true** Never True
2) The food that {I/we} bought just didn’t last, and {I/we} didn’t have enough money to get more food	229/1227	19 %	522/2432	21 %	
3) {I/we} couldn’t afford to eat balanced meals	198/1227	16 %	450/2432	19 %	
If an affirmative (often or sometimes true) response to questions 1, 2 or 3, then:					
4) Did {you/you or other adults in your household} ever cut the size of your meals or skip meals because there wasn’t enough money for food?	140/312	45 %	278/695	40 %	**Yes** No
5) Did you ever eat less than you felt you should because there wasn’t enough money to buy food?	134/312	43 %	278/694	40 %	
6) Were you ever hungry but didn’t eat because you couldn’t afford enough food?	84/312	27 %	164/695	24 %	
7) Did you lose weight because you didn’t have enough money for food?	51/310	16 %	98/694	14 %	
If an affirmative (Yes) response to any question 4–7, then:					
8) Did {you/you or other adults in your household} ever not eat for a whole day because there wasn’t enough money for food?	33/160	21 %	77/349	22 %	**Yes** No
If an affirmative (Yes) response to questions 4 or 8, then:					
9) How often did {you/you or other adults in your household} cut the size of your meals or skip meals?	110/140	79 %	220/278	79 %	**Almost every month** **Some months but not every month** Only 1 or 2 months
10) How often did {you/you or other adults in your household} ever not eat for a whole day because there wasn’t enough money for food?	28/33	85 %	59/77	77 %	

*n* 1227 Veterans and *n* 2432 non-Veterans. Questions refer to all household members, not just NHANES participants. Refused and do not know responses were excluded. Bold responses are considered affirmative for the classification of marginal, low and very low food security.

See Table 1 for all ten US FSSM questions and response options. For the first three questions, responses of ‘often true’ and ‘somewhat true’ were combined and categorised by NHANES as affirmative (yes) response, and ‘never’ was categorised as an answer of no. If no affirmative responses were selected to the first three questions, indicating high/full food security, then the remaining seven questions were not administered. However, an affirmative response to any of the first three questions prompted the remaining seven questions. For these questions (4–10), NHANES categorised responses of ‘yes’, ‘almost every month’ and ‘some months but not every month’ as affirmative. For this study, affirmative responses (≥ 1) to the first three questions indicated ‘food insecure’ (i.e. marginal, low and very low food secure). Additionally, responses of ‘refused’ or ‘did not know’ to any question were excluded from analysis (see Table 1).

Veterans, 18 years of age and older, were matched 1:2 to non-Veterans on self-reported age (±10 years), race/ethnicity, sex (male/female) and education. Race/ethnicities were categorised as the following: Hispanic (Mexicans and non-Mexican Hispanics), non-Hispanic Asian, non-Hispanic Black, non-Hispanic White and ‘Other’ race (including multi-racial). Education was categorised as the following self-reported categories: (1) less than high school graduate, (2) high school graduate/GED or equivalent, (3) some college or associates degree or (4) college graduate. If non-Veterans were unable to be matched for all matching criteria to Veterans, these non-Veterans were excluded from the analyses. We were unable to match 22 Veterans 1:2 with non-Veterans for all matching criteria; therefore, these Veterans were matched 1:1 with non-Veterans, resulting in inclusion of 1227 Veterans and 2432 Non-Veterans.

### Study variables

The prevalence of several chronic conditions was explored. The BMI variable was used to classify obesity using a cut point of ≥ 30 kg/m^2^. Diabetes was classified from variables of self-reported history of use of insulin or ‘diabetic pills to lower blood sugar’, and previous diagnosis of diabetes or a measured HbA1c ≥ 6·5 %. CVD was classified from a self-reported history of angina, CHD, myocardial infarction or stroke. Depression was classified based upon participant answers to a nine-item depression screening instrument^(19)^, which included questions related to: having little interest in doing things, feeling down, trouble sleeping or too much sleep, feeling tired or having little energy, loss of appetite or overeating, feeling bad about oneself, trouble concentrating, moving or speaking slowly or too fast and feelings of being better off dead. Response categories of ‘not at all’, ‘several days’, ‘more than half the days’ and ‘nearly every day’ were given a point ranging from 0 to 3, respectively. Scores for each question were added, and those with aggregate scores ≥ 10 were classified as depressed^(19)^.

Healthcare utilisation, access to care and poverty risk data also were examined. Healthcare utilisation was categorised as the number of self-reported visits (no visits, 1–3 visits, 4–9 visits or 10+ visits) an individual had ‘seen a doctor or other healthcare professional about their health at a doctor’s office, a clinic or some other place’ (not including hospitalisations overnight, visits to hospital emergency rooms, home visits or telephone calls) in the past 12 months. To examine poverty risk, we utilised the ratio of family income to poverty variable to categorise those having scores < 5 as ‘low income’ (income close to or at poverty level) and those with scores of 5+ as ‘high income’ (income at least five times greater than poverty level).

### Statistical analyses

Preliminary data cleaning was performed in R (R version 4.0.4), while all weighted data exploration and analysis were performed in SAS university (9.04.01M6P11072018). Survey means were obtained for continuous variables, while survey frequencies were used for all categorical variables. Survey logistic regression was used in determining predictors of the association between being a Veteran and adult food security, as well as to examine the relation of potential covariates (factors that modify the relationship) to this relationship. The model for the logistic regression was built beginning with a full model including all covariates. A backward stepwise approach was used in determining a best model by removing the covariates with the highest *P* values, one at a time, until all independent variables in the model had a *P* value less than 0·2. Covariates were selected a prior due to prior literature suggesting their influence on food security^(12–15)^. Covariates under consideration include age, sex, race/ethnicity, education, ratio of family income to poverty, healthcare utilisation, overnight stay in a hospital, obesity, diabetes, hypertension, CVD and depression. Age was analysed as a continuous variable, but all other covariates were analysed as categorical variables as outlined above and in Table 2. OR, CI and two-tailed *P* values were reported.

**Table 2:** Demographic characteristics of adults by Veteran status from National Health and Nutrition Examination Survey (NHANES) 2011–2016

	Veteran (*n* 1227)		Non-Veteran (*n* 2432)		*P*
	*n*	%	*n*	%	
Adult food security					0·11
High	915	78·53	1737	80·08	
Marginal	124	8·80	261	7·87	
Low	83	4·67	230	6·04	
Very low	105	8·00	204	6·01	
Sex					0·25
Female	103	9·76	206	8·33	
Male	1124	90·24	2226	91·67	
Race/ethnicity					0·08
Hispanic	165	6·57	329	6·59	
Non-Hispanic Asian	28	0·88	56	0·67	
Non-Hispanic Black	368	12·58	730	11·07	
Non-Hispanic White	617	75·53	1221	78·85	
Other race – including multi-racial	49	4·44	96	2·81	
Education					0·83
Less than high school	149	8·52	297	7·50	
High school	295	20·70	584	21·81	
Some college or advanced degree	474	39·36	938	38·60	
College graduate	309	31·42	613	32·08	
Obesity (BMI ≥ 30 kg/m^2^)					0·01
Yes	476	43·50	878	37·75	
No	689	56·50	1453	62·25	
Diabetes					0·01
Yes	297	19·56	481	15·77	
No	930	80·44	1951	84·23	
CVD					< 0·001
Yes	221	15·78	315	10·20	
No	1006	84·22	2117	89·80	
Hypertension					< 0·05
Yes	661	48·75	1188	44·14	
No	566	51·25	1244	55·86	
Depression					0·68
Yes	87	5·67	162	6·12	
No	1016	85·31	2014	85·80	
Missing	124	9·02	256	8·07	
Limited physical function					< 0·001
Yes	66	5·03	109	3·18	
No	585	47·21	941	37·52	
Missing	576	47·77	1382	59·30	
Healthcare utilisation					< 0·05
None (0 visits)	140	12·34	402	15·88	
1–3 visits	538	46·96	1109	47·51	
4–9 visits	440	32·78	770	31·12	
10+ visits	109	7·93	151	5·49	
Overnight hospital stay					0·03
Yes	171	11·83	296	9·34	
No	1054	88·17	2135	90·66	
Ratio of family income to poverty					0·4
Low income	975	71·12	1928	68·77	
High income	252	28·88	504	31·23	
Age (years)					
Mean	56·55		52·29		< 0·001*
sd	0·65		0·52		

*n* 1227 Veterans and *n* 2432 non-Veterans included in the analysis. NHANES data collected during 2011–2012, 2013–2014 and 2015–2016 were pooled for analyses. N are unweighted; percentages (%) are weighted. Continuous variables (age) reported as means and standard deviations, and categorical variables as percentages.

* *P* value determined by logistic regression; all other *P* values are calculated from Pearson’s Chi-Squared test.

## Results

Demographic characteristics between Veterans and non-Veterans are described in Table 2. By design, sex, ethnicity/race and education were similar between Veterans and non-Veterans, with the study population being 92 % male, ∼50 % non-Hispanic White and ∼30 % non-Hispanic Black, and the majority completing at least some college (∼37 %) or being a college graduate (25 %). However, Veterans (mean age: 57 years) were slightly (∼4 years) older than non-Veterans (*P* < 0·001). Regarding chronic conditions, Veterans were more likely to have obesity (44 % *v*. 38 %), diabetes (20 % *v*. 16 %) and CVD (16 % *v*. 10 %) (all *P*s < 0·01), respectively, but had similar rates of depression as non-Veterans (6 %). Veterans also were more likely than non-Veterans to self-report utilising health care and having a prior overnight hospital stay in the last year (all *P*s < 0·05). The prevalence of reporting a ‘low income’ was similar between groups (∼79 %).

Regarding food security status, the proportion of Veterans and non-Veterans reporting high (Veterans *v*. non-Veteran: 79 % *v*. 80 %), marginal (9 % *v*. 8 %), low (5 % *v*. 6 %) and very low (8 % *v*. 6 %) food security was similar between groups (*P* = 0·11). Additionally, the questions analysed from the US FSSM revealed similar results with regard to all ten questions (see Table 1): (1) worrying about running out of food (Veterans *v*. non-Veterans: 22 % *v*. 25 %, *P* = 0·26), (2) purchasing food that did not last, and not having money to get more (19 % *v*. 21 %, *P* = 0·13) and (3) an inability to afford balanced meals (16 % *v*. 19 %, *P* = 0·35). In those who were asked the remaining seven questions (4–10) due to selecting an affirmative response to the prior three questions, responses related to whether money influenced the following eating pattern decision also did not differ between groups: (4) had to cut size or skip meals (45 % *v*. 40 %, *P* = 0·15), (5) ate less than should (43 % *v*. 40 %, *P* = 0·55), (6) hungry but did not eat (27 % *v*. 24 %, *P* = 0·26), (7) lost weight due to lack of money for food (16 % *v*. 14 %. *P* = 0·26) and (8) did not eat for a whole day (21 % *v*. 22 %, *P* = 0·71). Additionally, there was no significant difference in how often adults (9) cut the size of meals or skip meals (79 % *v*. 79 % *P* = 0·92) or (10) did not eat for a day (85 % *v*. 77 %, *P* = 0·44).

Using univariate analyses (Table 3) to compare the odds of having high *v*. the combination of marginal, low and very low food security, we found that Veterans tended to be less likely to have high food security compared with non-Veterans (OR: 0·82 (95 % CI 0·66, 1·02), *P* = 0·07). We also found a significant relationship between several of our predetermined covariates and food security status. Non-Hispanic Black (OR: 0·53 (95 % CI 0·41, 0·69), *P* < 0·001) and other races including multiracial (OR: 0·55 (95 % CI 0·34, 0·88), *P* = 0·01) were less likely to have high food security compared with non-Hispanic White individuals. Completion of education less than a high school degree (OR: 0·37 (95 % CI 0·23, 0·61), *P* < 0·001), high school graduate or GED degree (OR: 0·39 (95 % CI 0·26, 0·57), *P* < 0·001) or some college or associates degree (OR: 0·47 (95 % CI 0·35, 0·63), *P* < 0·001) were less likely to have high food security compared with college graduates. Individuals with depression were less likely to have high food security compared with those not reporting depression (OR: 0·37 (95 % CI 0·26, 0·53), *P* < 0·001). Those with high income were more likely to have high food security compared to those with low income (OR: 6·60 (95 % CI 4·06, 10·75), *P* < 0·001). Additionally, the odds of having high food security increased for each year of age (OR: 1·03 (95 % CI 1·03, 1·04), *P* < 0·001). No other covariates were found to be significantly associated with food security status (Table 3).

**Table 3:** Association of Veteran status with prevalence of food security from National Health and Nutrition Examination Survey (NHANES) 2011–2016

	OR	95 % CI	*P*
Veterans (ref = no)			
Yes	0·82	0·66, 1·02	0·07
Sex (ref = female)			
Male	1·20	0·83, 1·74	0·33
Ethnicity (ref = White)			
Hispanic	0·72	0·49, 1·05	0·09
Non-Hispanic Asian	1·35	0·59, 3·12	0·47
Non-Hispanic Black	0·53	0·41, 0·69	< 0·001
Other races including multiracial	0·55	0·34, 0·88	0·01
Education (ref = college graduate)			
Less than high school	0·37	0·23, 0·61	< 0·001
High school	0·39	0·26, 0·57	< 0·001
Some college or AA degree	0·47	0·35, 0·63	< 0·001
Obesity (ref = no)			
Yes	0·91	0·72, 1·16	0·44
Diabetes (ref = no)			
Yes	0·80	0·63, 1·03	0·09
CVD (ref = no)			
Yes	0·75	0·53, 1·07	0·11
Hypertension (ref = no)			
Yes	0·78	0·59, 1·04	0·08
Depression (ref = no)			
Yes	0·37	0·26, 0·53	< 0·001
Missing	0·76	0·56, 1·03	0·08
Healthcare utilisation (ref = 0 visits)			
1–3	1·32	0·89, 1·95	0·17
4–9	0·90	0·61, 1·34	0·60
10 or more	0·78	0·48, 1·26	0·31
Overnight hospital stay (ref = no)			
Yes	0·98	0·68, 1·41	0·92
Ratio of family income to poverty (ref = low)			
High (5 or greater)	6·60	4·06, 10·75	< 0·001
Age	1·03	1·03, 1·04	< 0·001

*n* 1227 Veterans and *n* 2432 non-Veterans included in the analysis. NHANES data collected during 2011–2012, 2013–2014 and 2015–2016 were pooled for analyses. Univariate logistic regression determined OR, CI and *P* value. Food security status was defined as high food security *v*. food insecurity (combination of marginal, low and very low security).

The odds of Veterans having high food security (*v*. combination of marginal, low and very low food security) after controlling for *a priori* factors (age, sex, race/ethnicity, education, ratio of family income to poverty, healthcare utilisation, overnight stay in a hospital, obesity, diabetes, hypertension, CVD and depression) when presented overall and when stratified by sex, ethnicity and education are described in Table 4. Overall, after controlling for covariates, Veterans tended to be less likely to have high food security compared with non-Veterans (OR: 0·81 (95 % CI 0·65, 1·01), *P* = 0·06). Further, we found that non-Hispanic White Veterans were significantly less likely to experience high food security compared with non-Veterans in adjusted analyses (OR: 0·72 (95 % CI 0·55, 0·95), *P* = 0·02). Similar results were found when examining education where Veterans completing some college, but not having graduated college, were less likely to experience high food security compared with non-Veterans in adjusted analyses (OR: 0·71 (95 % CI 0·50, 0·99), *P* < 0·05). The risk of food insecurity (marginal, low and very low food security) did not differ by Veteran status when adjusted for covariates and stratified by any other category of race/ethnicity, education or sex.

**Table 4:** Association of Veteran status and food security prevalence when stratified by sex, ethnicity and education in National Health and Nutrition Examination Survey (NHANES) 2011–2016

	OR	95 % CI	*P*
Veterans (ref = no)			
Yes	0·81	0·65, 1·01	0·06
Sex			
Male	0·86	0·68, 1·07	0·17
Female	0·63	0·33, 1·21	0·16
Ethnicity			
Hispanic	1·24	0·74, 2·09	0·41
Non-Hispanic Asian	2·13	0·76, 5·96	0·13
Non-Hispanic Black	1·13	0·85, 1·50	0·40
Non-Hispanic White	0·72	0·55, 0·95	0·02
Other Race – including multi-racial	1·14	0·41, 3·16	0·80
Education			
Less than high school	1·72	0·89, 3·32	0·11
High school	0·77	0·49, 1·21	0·25
Some college or AA degree	0·71	0·50, 0·99	< 0·05
College graduate	0·88	0·48, 1·59	0·66

*n* 1227 Veterans and *n* 2432 non-Veterans included in the analysis. NHANES data collected during 2011–2012, 2013–2014 and 2015–2016 were pooled for analyses. Adjusted logistic regression stratified by sex, ethnicity and education determined OR, CI and *P* value. Food insecurity defined as the combination of the marginal, low and very low food security. Analyses adjusted for age, sex, race, education, obesity, diabetes, CVD, hypertension, depression, healthcare utilisation, overnight stay in a hospital and ratio of family income to poverty.

## Discussion

With Veterans often being of poorer health than non-Veterans^(20)^ and with food insecurity increasing the risk for poor health outcomes^(14)^, it is critical to identify the prevalence and socio-economic and biomedical determinates of food insecurity in this vulnerable and growing population. This study determined that the prevalence of food insecurity (marginal, low and very low food security) was 21 % in Veterans participating in NHANES, which was comparable to non-Veterans (20 %). These results are similar to a prior study utilising NHANES data, but these prior analyses were limited to Veterans with children. Further, they found no significant association in food insecurity between Veterans with children and non-Veterans with children^(10)^. This contrasts with our current findings and previous findings, including only adults by others reporting that, after adjusting for unknown covariates, Veterans are at greater risk to live in food insecure households compared with non-Veterans^(8)^. Overall, our study supports prior findings that ethnicity/race, education, income, age and depression are important covariates^(12,15,21)^.

Our study identified several racial and ethnic groups (non-Hispanic Black, Hispanic, non-Hispanic Asian and multiple races) as being less likely to have high food security compared with non-Hispanic White individuals, further adding to the evidence that racially and ethnically minoritised individuals are at increased risk for food insecurity within the general population^(12,21)^. On the other hand, for Veterans, food security status and racial disparities may not follow this same pattern. After controlling for covariates, we found that non-Hispanic White Veterans were significantly less likely to experience high food security compared with non-Hispanic White non-Veterans. This could be attributed to several underlying factors, including that non-Hispanic White males make up 78 % of the Veterans population and are less likely to have a high school diploma when compared with other Veteran race/ethnicities and their non-Veteran counterparts^(22)^ Additionally, Veterans with or without a high school diploma are more likely to be disabled compared with similar non-Veterans^(23)^. Therefore, being at increased risk for less education and more disabilities could potentially lower income and may place non-Hispanic White Male Veterans at a greater risk for food insecurity, compared with non-Veterans. It should be mentioned that a high school diploma/GED is a current requirement to enlist in the military; however, this educational requirement has not always been the case. Previous reports suggest that 15 % of Veterans aged 55– 64 years, 24 % aged 65–74 years and 49 % aged 75 years and older did not have a high school diploma upon enlistment, which can be attributed to the military draft between 1940 until 1973 (during peacetime and periods of conflict) when military service was obligatory^(22)^. These data likely account for the lower education classification within the Veteran cohort. Thus, food security comparison data should be interpreted with the above underlying factors in mind.

In contrast, military service can increase educational attainment and income for historically excluded groups when compared with similar non-Veterans^(24–27)^. For racially and ethnically minoritised individuals from disadvantaged backgrounds, military service has been shown to improve occupational skills, expand social network^(24,25,27,28)^ and provide educational financial assistance from the G.I. Bill^(29)^, which potentially lowers the risk for food insecurity, compared with similar non-Veterans. Other studies have identified additional Veteran specific socio-economic risk factors that increase the risk of food insecurity. This includes a 2021 USDA report on food insecurity among working-age Veterans, which identified those who are younger, have a serious mental illness or recently discharged from active duty, as having a food insecurity prevalence rate that is more than double that of non-Veterans^(8)^. The identification of socio-economic risk factors specific to the Veteran population is important because it allows for monitoring of those at higher risk and potential interventions on modifiable risk factors.

Studies examining the prevalence of food insecurity among Veterans are inconsistent, with results ranging from 6 % to 25 %^(9,30)^. Varying results are reported between studies that compare prevalence between Veterans and non-Veterans, with studies reporting the prevalence of food insecurity in Veterans to be more^(8)^, less^(9,11)^ and comparable^(10)^ (including our current analysis) to non-Veterans. Further, methodological differences between studies limit our ability to directly compare our results with those of other studies. One important difference includes how food security status is defined.

The criteria used to define food security status are key when interpreting and comparing results as the definition alone could skew the results. Food security status classification schemes vary across studies, with many defining food security status based on a continuum of high, marginal, low and very low food security. Numerous studies that use this continuum, including the US government, classify ‘food secure’ as high and marginal food security and ‘food insecure’ as low and very low food security^(10,14)^. Additionally, some define food security status based only on very low food security compared with the combination of high, marginal and low food security, while some simply define food security status based on a ‘yes’ response to one question^(11,31,32)^. Classifying marginal food security (households that experience problems or anxiety, at times, with accessing adequate food^(14)^) in the same category as high food security has the potential to underestimate the prevalence and impact of adverse health outcomes associated with exposure to not consistently having access to adequate food. Accumulating evidence suggests that adults with marginal food security are at increased risk for developing a chronic disease (i.e. cardiovascular, hypertension, diabetes^(14)^, metabolic syndrome^(33)^), more likely to report poor health, have multiple chronic diseases^(14)^ and at greater risk of impaired nutrition^(34)^ compared with food secure adults. Furthermore, from a public health perspective, categorising marginal food security as food secure limits the ability of policymakers to assess the effectiveness of nutrition assistance programmes in reducing and preventing food deprivation. Therefore, marginal food security was included as a risk indicator for food insecurity in this study. Thus, adult household food security status was defined as high food security *v*. food insecurity (combination of marginal, low and very low security).

In addition to the various classification schemes, studies examining food security status in Veterans use a wide variation of surveys, sample populations and covariates^(35)^. A recent review by Cypel *et al*. reported that twenty out of twenty-one articles examining food insecurities among Veterans used different data sources (nationwide population-based surveys, Veterans Health Administration (VHA) surveys, focus groups and pilot studies), baseline characteristics and terminology to define ‘food insecurity’^(35)^. The design structure of both nationwide population and Veteran Affairs (VA)-based surveys are fundamentally limited in their ability to fully capture or represent the entire US Veteran population. Nationwide population-based surveys, structured to sample civilian populations, often have an imbalance of Veteran to non-Veterans represented, and the Veterans captured may not fully represent the Veteran population (sex, race, deployment, combat exposure, rank, service era, branch, length of service, etc.) as a whole. As evident by Miller *et al*., who used data from the Current Population Survey, only 6·5 % of the sample in the published report consisted of Veterans who served post-Vietnam^(11)^. On the other hand, VA surveys only include Veterans who receive care through VHA. With only 50 % of Veterans receiving care from the VHA^(36)^, these surveys may not be representative of the entire Veterans population. Additionally, sample populations/baseline characteristics across studies vary from Veterans *v*. non-Veterans, Iraq and Afghanistan Veterans, Women Veterans, Minneapolis Veterans, Veterans with HIV, Veterans with children, etc., with target populations ranging from 18 to 85 years of age. Covariates also tend to differ across most studies^(11,30–32)^. Like our study, many controlled for age, sex, race/ethnicity, education, depression and income^(10,11,31)^. Other covariates used in studies include, but are not limited to, the presence of immigrants in the home, marital and employment status, tobacco and alcohol use, military rank, trauma exposure and various health conditions^(11,30–32)^. With no standardised methodology established to examine food insecurity in Veterans, our ability to generalise and interpret results between studies is limited.

Due to the availability of NHANES data, our results are limited to the time frame of 2011–2016. The evaluation of affirmative responses to the first three independent questions of the US FSSM found ∼20 % of Veterans reported worrying about food running out, not having enough money to get more food and affording balanced meals. Additionally, of these food insecure Veterans, over 40 % reported that they had to cut the size or skip meals and/or eat less than they felt they should have because there was not enough money to buy food. Furthermore, though the prevalence of affirmative responses to the question about not eating for a day because there was not enough money for food did not differ statically between food insecure Veterans (85 %) and non-Veterans (77 %), these results should be interpreted in light of the small sample of respondents for this question (only 33 Veterans and 77 non-Veterans) and in regard to their clinical relevance. These results are alarming and intensify the need for access to resources available to alleviate food insecurity in Veterans. However, it must be noted that efforts to improve food security status in Veterans are constantly evolving. As a result of a 2015 Congressional briefing on Veteran food security, the VHA formed ‘The Ensuring Veteran Food Security Workgroup’ to address this issue^(37)^. This workgroup has partnered with government and non-profit organisations to: examine food insecurity issues, identify Veterans at risk, promote interprofessional care and support the expansion of VA facilities involved in on-site or mobile food panties (Veterans Pantry Pilot)^(37)^, which currently serve more than 40 000 Veterans at seventeen VA locations^(38)^. Additionally, in 2017, the VHA implemented a one question food insecurity screener, which is completed on all non-institutionalised Veterans receiving care^(39)^. The integration of the food insecurity screener is a critical step to identify and provide assistance to vulnerable Veterans. As of 2020, over 5 million Veterans have been screened^(39)^.

In addition to VA programmes and initiatives, both federal (i.e. Supplemental Nutrition Assistance Program (SNAP)) and community (i.e. food panties) resources are available to food insecure Veterans^(39)^. However, many individuals may not be utilising these resources and programmes. For example, 59 % of eligible Veterans do not participate in the SNAP^(39)^. Several barriers may limit Veteran participation, including lack of knowledge of available resources, stigma surrounding programme utilisation, pride and beliefs about self-reliance that may have developed during military service^(40)^. Additionally, many may be ineligible for federal nutrition programmes based on income limits. The State of Hunger in American 2016 reported that two-thirds of food insecure older adults have income over the federal poverty line, thus not qualifying for SNAP^(41)^. This scenario could also affect some food insecure Veterans; however, that has yet to be determined.

### Limitations

There are several limitations to the current study. First, the cross-sectional study design and self-report response bias prevent any conclusion from being drawn about cause and effect and how the prevalence of food security may change over time. Due to limitations in data collected by NHANES, other potential covariates (i.e. combat exposure, service era, occupation during military service and rural *v*. urban community location) that may modify the relationship between Veteran status and food security could not be explored. This is an area for future studies to explore. Additionally, the analyses include both fixed and modifiable covariates and do not allow for determination of temporal direction of the effect, rather they only allow for determination of an association between the variables of interest. Furthermore, Veterans NHANES is a nationwide general population survey that does not seek to sample Veterans. Thus, selection bias may have resulted in an imbalance of Veteran to non-Veterans and may not fully represent the entire Veteran population. However, the large number of non-Veterans represented allowed us to closely match Veterans to non-Veterans. Finally, the small sample size of the food security comparison data (see Table 1) increases the likelihood of a type II error, which decreases the power of the comparison. Therefore, the food security comparison data may not be generalised to the entire Veteran population.

## Conclusion

In order to develop interventions better suited to address the unique needs of the Veteran population, it is essential to understand the risk and the socio-economic factors associated with food security status in Veterans compared with non-Veterans. After adjusting for covariates, we found that Veterans are less likely to experience high food security compared with non-Veterans. This study supports previous research that identified ethnicity and/or race, education, income, age and depression as important covariates when examining food security status in the Veteran population. Additionally, it adds to the literature by highlighting ethnicity and level of education as important socio-economic determinates of food security status in Veterans.
